# Patient and public involvement and engagement to improve impact on antimicrobial resistance

**DOI:** 10.1038/s41467-024-55410-8

**Published:** 2025-01-25

**Authors:** Emily Scott-Dearing, Vanessa Carter, Michael Corley, Philip Mathew, Ara Darzi

**Affiliations:** 1 Fleming Initiative, Institute for Global Health Innovation, Imperial College London, South Kensington Campus, London, UK; 2 The AMR Narrative, Suite 3, Peel House, 30 The Downs, Altrincham, Cheshire, UK; 3 British Society for Antimicrobial Chemotherapy, 53 Regent Place, Birmingham, UK; 4 AMR Global Coordination and Partnership department, World Health Organization, Avenue Appia 20, Geneva, Switzerland

**Keywords:** Public health, Patient education, Infectious diseases, Culture, Society

## Abstract

A step change is required in citizen awareness of antimicrobial resistance (AMR). The authors propose we go further still, directly engaging and involving patients and public in scoping, testing and scaling solutions to this global health crisis.

## Teaser

A step change is required in citizen awareness of the global antimicrobial resistance (AMR) health crisis. But we must move beyond awareness-raising to action, directly engaging and involving patients and public in scoping, testing and scaling solutions to ensure their adoption and impact.

The impacts of antimicrobial resistance (AMR) on society are serious, growing and inequitable. On current trajectory antimicrobial-resistant infections will kill 1.9 million people a year by 2050, mostly in Low- and Middle-Income Countries (LMICs). One in five deaths attributable to AMR occur in children under five years old and AMR mortality among adults aged 70+ is rapidly accelerating^1^. Overuse and misuse of antimicrobials are driving the declining efficacy of this cornerstone of modern medicine^2^, which also correlates with poor access to water, sanitation and hygiene, including in contexts of disaster and instability.

Wide-ranging research and innovation are underway to contain, control and mitigate AMR. But like the climate crisis, science cannot solve this alone. AMR is not simply a scientific problem requiring scientific solutions, but a societal issue in which individual behaviours and choices^3^ play a significant part in both its aetiology and mitigation. Improvements in the status quo will be difficult to achieve, and impossible to sustain, without ensuring solutions are responsive to the needs of the public. Structural barriers and inequalities around access to quality assured healthcare delivery also need to be highlighted and addressed.

## Raising awareness to promote behaviour change

Slowing AMR requires a step change in global citizen awareness of its causes and solutions, as a pre-requisite for collective behaviour change by public, practitioners and policy makers. This requires effective and wide-ranging communication of the issue, and individuals being made aware when their ill health may be caused by AMR. Reporting of AMR to patients is likely insufficient. In addition, framing of the issue for the public has been technical and fragmented to date and public awareness and understanding of AMR is low and variable^4^. Encouragingly the importance of engagement is now being recognised internationally^5^, including at the United Nations, where a participatory approach was acknowledged in the 2024 Political Declaration of the High-level Meeting on Antimicrobial Resistance, and in National Action Plans, although resourcing and adoption of the latter is inadequate. The burden of AMR is felt most heavily in LMIC’s but whilst there are AMR engagement success stories in these regions, these are not wide-reaching or sustained, the specificity and context-dependency of this work can make it difficult to evaluate and scale^6^, and there is likely less resource applied to delivering and documenting such activity in LMIC settings.

Comprehensive themes, effective for engaging the public across multiple countries have been identified^7^. One key approach is to humanise the issue by describing AMR’s personal impact on individuals, including patient survivors and those who have lost a loved one – recognising that numbers and statistics can be less effective compared to narrative evidence in improving understanding and eliciting preventative behaviours^8^. This is a universal issue that can affect anyone, and we need to forge an emotional connection with people. The Bureau of Investigative Journalism’s hard-hitting photojournalism exhibition highlights the devastating impacts of AMR on the most vulnerable and gives immediacy to a slowly unfolding catastrophe. Patient advocacy groups like the Sepsis Alliance (USA), The AMR Narrative (UK), MRSA Action Aid (UK) and TB Proof (South Africa); and the World Health Organisation’s (WHO) campaign called AMR is invisible. I am not, demonstrate the power of foregrounding the experience of AMR patient survivors and carers. WHO’s Task Force of AMR survivors’ members who feature in that campaign represent diverse medical conditions, socioeconomic backgrounds and include members from HICs, LMICs and conflict zones. They have identified a range of opportunities for meaningful patient and carer engagement on AMR, within and beyond research^9^.

## Catalysing innovative collaborations to maximise impact

Those of us seeking to drive citizen behaviour change to tackle AMR need to build a shared sense of motivation and urgency between those working on AMR and those just learning about it for the first time. To maximise impact, we must forge novel partnerships with those not currently engaged with AMR, including across the cultural, creative, advocacy and entertainment sectors. Drawing on their engagement, storytelling and campaigning skills will enable us to connect our knowledge and call for action most effectively with the widest possible audiences. Developing and testing messaging and framing with those audiences will be essential, as the creative agency Ogilvy and Mather Bangkok did for their low budget, high-impact Smoking Kid anti-smoking campaign for the Thai government in 2014.

The touring exhibition ‘Superbugs: the fight for our lives’ by the UK’s Science Museum Group has reached an audience of 1.5 million via science centres and museums across India and China. WHO’s Fides network and YouTube Health each mobilise clinician content creators to deliver high quality public health messaging, including on AMR, on social media to their existing subscribers that total in the tens of millions. Lifeline The Musical has brought true stories of AMR’s impact to theatre audiences in UK and USA, integrating local citizens into their casts to heighten the authenticity and power of their message, and develop their agency around the issue.

## Moving beyond awareness to patient and public involvement

But raising awareness of AMR will not be enough. The solutions we develop to address this crisis must be relevant, valued and trusted by all, to ensure their widespread implementation and impact. We must move beyond awareness to action, recognising the benefits of directly engaging and involving the public in scoping, testing and scaling solutions^10,11^ – be they prevention measures, behavioural nudges, diagnostics, new treatments, agricultural and environmental protocols, or legislation – so that these solutions are ready for implementation and unintended consequences are avoided.

The terminology describing active involvement of citizens in research, healthcare and policymaking varies as much as the methods by which it is achieved. It goes by many names including patient and public involvement, public engagement, community engagement and participation. Involvement opportunities can be broad but relatively shallow – for example thousands of so-called citizen scientists each contributing a data point or analysis to one step of a research method. Or they can be deeper and sustained – for example, involvement of patient advocates in all stages of study design and delivery, as co-designers of the research.

The case for the benefits of patient and public involvement has been championed for example by National Institute for Health Research (NIHR) in the UK (where it has been an embedded principal of Government health research strategy since 2006). They argue that this approach improves the quality of research, how it is designed, conducted and delivered^12^. Similarly, active involvement of citizens in (health) policymaking can correct misalignment between policymakers and the priorities and experiences of citizens, improving policy relevance and mobilising citizens as a lever for change^13^.

Much has been published to describe and guide best practice, although standardised approaches for measuring impact have been slower to emerge, limiting the published evidence base. Key principles from the UK’s National Co-ordinating Centre for Public Engagement, highly relevant to those tackling AMR, include designing your approach to patient and public involvement with a clear sense of its specific purpose, the people you want to involve and your context in mind. Also key to good practice is investing time to build trusting, long-term relationships between researchers and patients or public – even beyond the time frame of individual projects – to ensure the research is responsive to the local context and needs of the community, and to achieve genuine community involvement in and ownership of the research^14^.

## Enabling a culture where patient and public voices are valued

What would this look like for AMR? Citizens, including AMR patient survivors and community leaders, would be actively involved in shaping policy and research priorities and methodologies by including them at all possible stages of this work, including its governance. AMR research and policy questions would be explored with the public by incorporating them into a spectrum of engagement activities, from interactive exhibits to digital surveys and in-person structured consultations^15^.

What would it take to build a culture where the public voice is valued by those seeking to address AMR, through research, medical education, awareness raising, innovation or policy? First, recognition that the critical benefits outlined require a shift in practice and an acknowledgement that working in this way comes with additional demands. It takes extra time and resource; training, capacity building and mentoring including for and with patient advocates; sharing of learning, methodologies and resources, to be adapted by local teams for local contexts. As per the climate emergency, public concern about an issue and democratic calls for action can be a powerful lever for policy change, complementing evidence generation and expert advocacy.

## Localising approaches to address this global health crisis

Drug-resistant infections do not respect national borders and populations across the world face a myriad of unevenly distributed AMR drivers and impacts. Solutions will require a global effort that recognises the wide disparities in systemic strength and resources. Tackling COVID-19 required global sharing of knowledge and resources, coordination of data and messaging, and communication from trusted sources to generate a shared sense of urgency and to achieve action with impact. Whilst challenges were apparent in disseminating accurate information and in the global coordination of responses, much was demonstrated and learned during the pandemic about what is possible, including online social listening strategies and working with local communities to adapt messaging. Now we need to do the same, more effectively and equitably, for AMR. That effort needs to be capable of flexing to address specific local, national and global challenges. Communications and proposed solutions need to be generously shared and devolved, adapted and delivered by local teams to suit the cultural context and meet local needs.
